# Evaluating drug-coated balloon versus drug-eluting stent for the treatment of small coronary artery disease: a meta-analysis and systematic review

**DOI:** 10.3389/fcvm.2026.1906701

**Published:** 2026-09-21

**Authors:** Yanjie Gao, Ming Lian

**Affiliations:** Emergency Internal Medicine Department, The Affiliated Hospital of Inner Mongolia Medical University, Hohhot, China

**Keywords:** coronary artery disease, drug-coated balloon, drug-eluting stent, meta-analysis, systematic review

## Abstract

**Objective:**

This meta-analysis aimed to compare the efficacy and safety of drug-coated balloons (DCB) vs. drug-eluting stents (DES) in patients with *de novo* small coronary artery disease.

**Methods:**

We systematically searched PubMed, Embase, Cochrane Library, and Web of Science from inception to March 18, 2024, for randomized controlled trials comparing DCB with DES in small-vessel coronary disease (reference vessel diameter ≤2.75 mm or ≤2.5 mm). Primary outcomes included nonfatal myocardial infarction, cardiac death, all-cause death, target lesion revascularization (TLR), and target-vessel revascularization (TVR). Pooled odds ratios (ORs) with 95% confidence intervals (CIs) were calculated using fixed- or random-effects models depending on heterogeneity. Certainty of evidence was assessed using the GRADE framework. The protocol was registered with PROSPERO.

**Results:**

Six unique RCT cohorts (eight publications) comprising 1,849 patients were included. No significant differences were observed between DCB and DES for nonfatal myocardial infarction (OR = 0.87, 95% CI: 0.55–1.39; *I*^2^ = 39%), cardiac death (OR = 1.38, 95% CI: 0.66–2.88; *I*^2^ = 0%; four trials), all-cause death (OR = 1.00, 95% CI: 0.59–1.69; *I*^2^ = 0%), TLR (OR = 1.03, 95% CI: 0.65–1.64; *I*^2^ = 25%), or TVR (OR = 0.76, 95% CI: 0.47–1.23; I^2^ = 34%). Subgroup analysis suggested a potential TLR benefit in very small vessels (≤2.25 mm), but this finding was exploratory with no significant interaction across subgroups. All outcomes were rated as moderate certainty, except TVR (low to moderate), with downgrading primarily due to imprecision and open-label design limitations.

**Conclusion:**

Current randomized evidence indicates that DCB provides comparable clinical outcomes to DES in patients with *de novo* small coronary artery disease. However, the limited number of trials and insufficient power for subgroup analyses underscore the need for larger, well-powered randomized studies to define the optimal strategy, particularly in specific patient and vessel subsets.

**Systematic Review Registration:**

https://www.crd.york.ac.uk/PROSPERO/view/, identifier CRD420261500541.

## 1 Introduction

Coronary artery disease involving small vessels (typically defined as a reference vessel diameter ≤2.75 mm) represents a frequent and clinically challenging presentation in interventional cardiology. Narrowing in these microvessels is a common angiographic finding and is associated with adverse outcomes, including higher rates of restenosis and repeat revascularization (1). Percutaneous coronary intervention (PCI) in small coronary arteries now constitutes 30%–40% of all PCI procedures, underscoring the growing clinical relevance of this anatomical subset and the need for optimized therapeutic strategies (1).

Despite its prevalence, the management of *de novo* small coronary artery disease lacks universally accepted guidelines, leading to variability in clinical practice (2). Drug-eluting stents (DES), while effective in larger vessels, face inherent limitations in small-caliber arteries, where their use has been associated with elevated restenosis rates, often attributed to increased shear stress, greater neointimal response, and suboptimal stent-to-vessel sizing (3–6). These challenges are compounded in diffusely diseased segments, where long stent lengths may further heighten the risk of stent thrombosis and restenosis.

Drug-coated balloons (DCBs) have emerged as a promising stent-free alternative, designed to overcome limitations inherent to both bare-metal and drug-eluting stents (3). The DCB platform combines a semi-compliant balloon with a matrix-encapsulated antiproliferative drug—most commonly paclitaxel—that is rapidly transferred to the vessel wall during balloon inflation. This mechanism enables local drug delivery without the permanent implantation of metallic scaffolds or durable polymers (4).

By avoiding a permanent implant, DCBs theoretically mitigate several stent-related drawbacks: they reduce late inflammatory responses, preserve physiological vasomotion, lower the risk of stent thrombosis, and may permit shorter durations of mandatory dual antiplatelet therapy (DAPT) (5). These characteristics make DCBs particularly appealing for small-vessel interventions, where stent deployment is often technically constrained and associated with higher rates of adverse vascular remodeling.

Although DCBs are well-established for the treatment of in-stent restenosis, their role in *de novo* small coronary lesions requires further validation. Current evidence remains limited and heterogeneous, with variations in study design, patient selection, procedural technique, and endpoint definitions confounding clear clinical interpretation. Moreover, in patients with multivessel disease, a hybrid revascularization approach—combining DES in larger proximal segments with DCBs in distal small vessels—has been proposed as a tailored strategy to optimize outcomes while minimizing metal burden (7).

Given the expanding use of PCI in small vessels and the ongoing uncertainty regarding the comparative effectiveness of DCB vs. DES, this systematic review and meta-analysis aims to synthesize the available randomized and comparative evidence to evaluate the efficacy and safety of DCBs relative to DES in patients with *de novo* small coronary artery disease.

## 2 Data collection and methodology

### 2.1 Literature retrieval

A thorough search was independently conducted by two researchers across multiple databases, including PubMed, Embase, Cochrane Library, and Web of Science. The search strategy employed a combination of keywords encompassing drug-coated balloons, drug-eluting stents, and coronary vessel diseases. This search was carried out from database inception until March 18, 2024, and was limited to publications in the English language. Ethical clearance was not required as this study relied solely on data obtained from previously approved original articles.

This study adhered to the rigorous guidelines outlined by the Preferred Reporting Items for Systematic Reviews and Meta-Analyses (PRISMA) for conducting systematic reviews and meta-analyses.

### 2.2 Inclusion criteria

Following the PICOS principle, literature inclusion was determined based on the following criteria: Population [patients with small coronary artery disease, precisely defined by a reference vessel diameter (RVD) threshold, typically ≤2.75 mm or ≤2.5 mm], Intervention (DCB), Comparison (DES), Outcome (nonfatal myocardial infarction, cardiac death, all-cause death, target lesion revascularization, target-vessel revascularization, among others), and Study Design (prospective randomized controlled trials only). Observational studies, including propensity-score matched comparative studies, were excluded to maintain methodological consistency and minimize heterogeneity arising from differences in study design. Exclusion criteria encompassed studies not meeting the intervention criteria, those with unclear surgical procedures, incomplete data, or those with a sample size less than 10. In instances where multiple publications were identified with the same clinical registration number, the most recent publication was selected.

### 2.3 Data extraction

Two authors independently conducted data extraction and eligibility assessment. Information extracted from original articles encompassed author details, publication year, country, intervention methods, sample size, population characteristics (ACS vs. stable presentations), study design, follow-up duration, and outcomes. When multiple publications originated from the same randomized controlled trial cohort, only the publication reporting the primary endpoint or the longest follow-up data was selected for each outcome to avoid double counting of participants. We specifically extracted key procedural and device variables, including RVD thresholds, lesion length limits, *de novo* vs. ISR inclusion, DCB drug type (paclitaxel vs. limus), DES generation, lesion preparation requirements, bailout stenting rates, and DAPT regimen/duration. A pre-designed Excel table facilitated this process, with any discrepancies resolved through consultation with a third researcher.

### 2.4 Evaluation of study quality

Study quality was assessed using the revised Cochrane risk-of-bias tool for randomized trials (RoB 2). This systematic evaluation covered five essential domains: the randomization process, deviations from intended interventions, missing outcome data, measurement of the outcome, and selection of the reported result, with justifications provided for each domain-level judgment. This thorough evaluation delved into various dimensions, scrutinizing aspects such as the randomness of sequence generation, concealment of allocation, blinding procedures for both participants and personnel, blinding of outcome assessment, completeness of outcome data, selective reporting of outcomes, and identification of any other potential sources of bias.

### 2.5 Statistical analysis

The study analyzed both categorical and continuous outcome variables. Categorical data were assessed using risk ratios (RR) and their corresponding 95% confidence intervals (CI), while continuous data employed standard mean differences (SMD) and their 95% CI. Heterogeneity between studies was evaluated using Cochran's *Q*-test, with *I*^2^ values of 0%, 25%, and 50% representing low, moderate, and high heterogeneity, respectively. To investigate potential sources of heterogeneity and ensure clinical transferability, we performed pre-specified sensitivity and subgroup analyses. These focused on critical variables including vessel diameter (very small <2.25 mm vs. borderline ≥2.25 mm), DCB drug platform (paclitaxel vs. limus), stent generation, and follow-up duration. Fixed-effects or random-effects models were employed based on the level of heterogeneity. To handle studies with zero events in one or both arms, a continuity correction of 0.5 was applied to all cells of the contingency table to enable inclusion in the pooled risk ratio calculations and avoid the exclusion of informative trials. Publication bias was explored using funnel plots when sufficient studies were available. All statistical analyses and graphical representations were performed using Stata software, with statistical significance set at a two-sided *p*-value < 0.05.

## 3 Results

### 3.1 Literature search overview

A comprehensive search yielded a total of 1,467 potentially relevant articles published between 2010 and October 2023. Following the removal of duplicates, 1,289 unique articles remained (972 in English and 317 in Chinese). Title and abstract screening led to the exclusion of 1,272 studies due to irrelevance, non-comparative design, or review formats. Subsequently, 17 full-text articles were evaluated, of which 8 were excluded due to missing data or mismatched diagnostic criteria. After removing duplicate reports from the same randomized controlled trial cohorts, six studies contributed data to the analysis of nonfatal myocardial infarction. Among 1,849 participants, nonfatal myocardial infarction occurred in 33 of 875 patients in the DCB group and 42 of 974 patients in the DES group (Figure 1).

**Figure 1:**
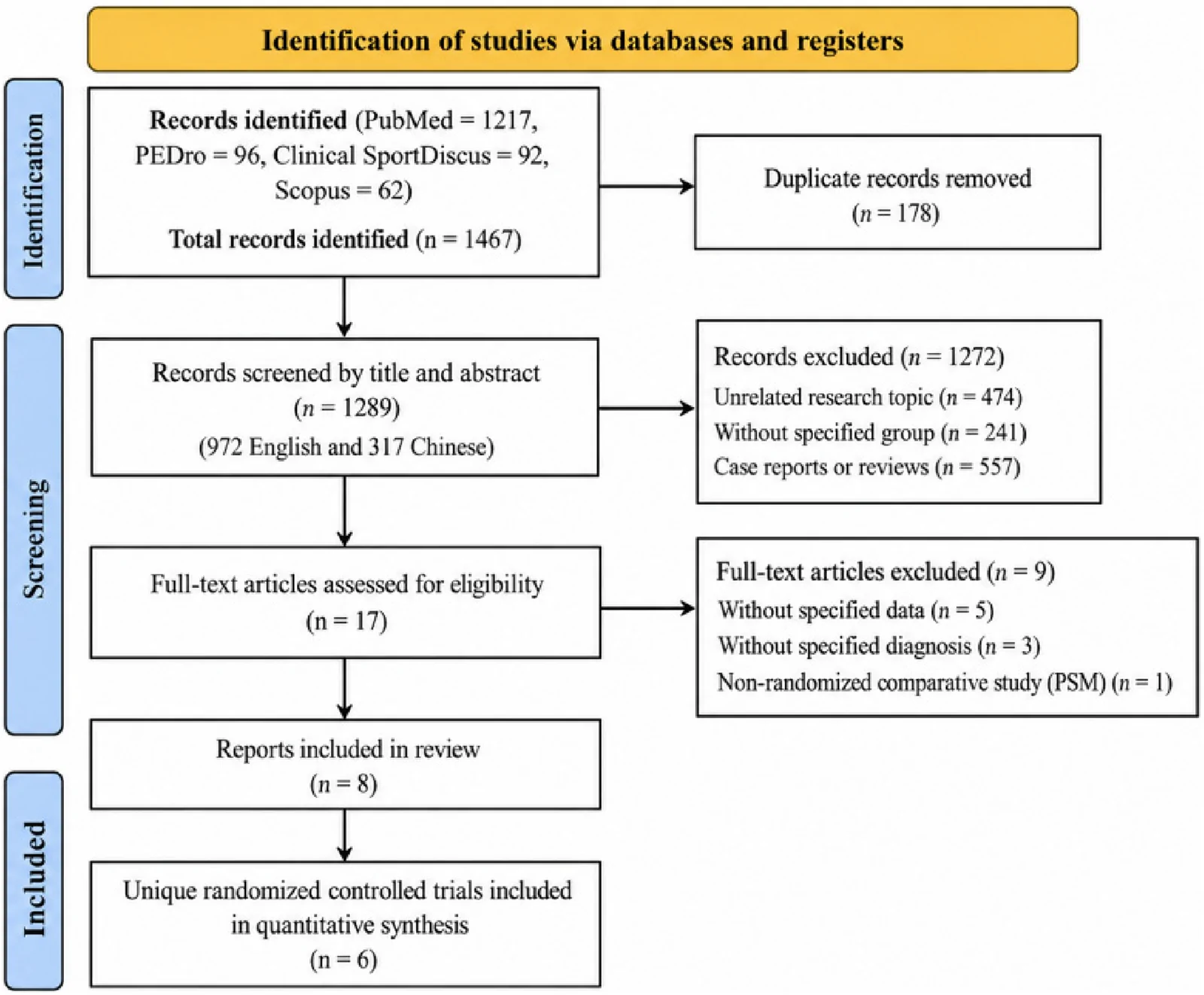
Flowchart of literature selection process.

### 3.2 Characteristics of included studies

Key characteristics of the six included RCTs are detailed in Table 1. Evaluated clinical endpoints across trials included nonfatal myocardial infarction, cardiac death, all-cause death, target lesion revascularization (TLR), and target-vessel revascularization (TVR). To account for procedural heterogeneity, specific endpoint definitions (e.g., peri-procedural vs. spontaneous myocardial infarction, clinically driven vs. protocol-driven TLR/TVR) and independent adjudication status are systematically detailed in Table 2.

**Table 1:** provides a comprehensive overview of the included trials, detailing the standardized definitions of small vessels (RVD thresholds) and key technique-level variables such as lesion preparation, drug types, and bailout stenting rates.

Authors	Time	Treatment Modalities (DCB vs. DES)	Drug-Eluting Stent (n)	Drug-Coated Balloon (n)	RVD Threshold	Lesion Length	Drug Type	Lesion Prep	Bailout Rate	DAPT	Outcomes
Cortese (8)	2010	DCB vs. DES	29	28	≤2.75 mm	≤20 mm	Paclitaxel	Pre-dilation	3.40%	6 months	a, b, c, d, e
Latib/BELLO (9)	2012	DCB vs. DES	92	90	≤2.75 mm	≤20 mm	Paclitaxel	Pre-dilation	3.30%	6 months	a,b, c, d, e
Sinaga (10)	2016	DCB vs. DES	163	172	≤2.75 mm	≤20 mm	Paclitaxel	Pre-dilation	2.30%	12 months	a, b,c, d,e
Sim (11)	2018	DCB vs. DES	200	87	≤2.5 mm	≤20 mm	Paclitaxel	Pre-dilation	5.70%	6 months	a, b, c, d,e
Jeger/BASKET-SMALL (12)	2018	DCB vs. DES	376	382	≤2.5 mm	≤20 mm	Paclitaxel	Pre-dilation	5.40%	6 months	a, b, c, d, e
Tang (13)	2018	DCB vs. DES	114	116	≤2.75 mm	≤20 mm	Paclitaxel	Pre-dilation	4.30%	6 months	a, b, c, d, e

RVD Threshold: Reference Vessel Diameter, typically ≤2.75 mm or ≤2.5 mm for small vessels.

Lesion Length: Where available, the maximum lesion length reported for the studies (≤20 mm in most studies).

Drug Type: Paclitaxel or Everolimus, as stated in the studies.

Lesion Prep: All studies involved pre-dilation of lesions.

Bailout Rate: The rate at which stent implantation was required during the procedure due to complications (reported as a percentage).

DAPT: Duration of Dual Antiplatelet Therapy, typically 6 months in the studies.

Outcomes: a: nonfatal myocardial infarction, b: cardiac death, c: all-cause death, d: target lesion revascularization, e: target-vessel revascularization.

**Table 2:** Endpoint definitions across included randomized controlled trials.

Study	Myocardial Infarction	Target Lesion Revascularization (TLR)	Target Vessel Revascularization (TVR)	Independent Adjudication	Definition of Events
Cortese (8)	Peri-procedural and spontaneous	Clinically driven	Protocol-driven	Yes	MI: defined as a rise in cardiac biomarkers (CK-MB, troponin); TLR: revascularization driven by ischemic symptoms or positive stress testing; TVR: revascularization of the target vessel based on angiographic criteria.
Latib (9)	Peri-procedural and spontaneous	Clinically driven	Protocol-driven	Yes	MI: increase in CK-MB or troponin levels with associated symptoms; TLR: clinically driven by symptoms or evidence of ischemia; TVR: protocol-driven when restenosis was confirmed by angiography.
Sinaga (10)	Peri-procedural and spontaneous	Clinically driven	Protocol-driven	Yes	MI: defined by elevation of troponin or CK-MB with chest pain; TLR: clinically driven based on angiographic or symptomatic indications; TVR: required when restenosis exceeded 50%.
Sim (11)	Peri-procedural and spontaneous	Clinically driven	Protocol-driven	Yes	MI: increase in cardiac biomarkers with or without symptoms; TLR: symptomatic revascularization with positive stress testing or clinical symptoms; TVR: restenosis identified through angiographic follow-up.
Jeger (12)	Peri-procedural and spontaneous	Clinically driven	Protocol-driven	Yes	MI: elevation of cardiac biomarkers (troponin/CK-MB); TLR: clinically driven based on symptoms or ischemic changes on imaging; TVR: protocol-driven when ≥50% stenosis was identified.
Tang (13)	Peri-procedural and spontaneous	Clinically driven	Protocol-driven	Yes	MI: confirmed by increased cardiac biomarkers with symptoms; TLR: clinically driven based on ischemic events or stress test findings; TVR: protocol-driven based on angiographic findings.

Peri-procedural MI: Defined as a rise in cardiac biomarkers (CK-MB, troponin) during or immediately after the procedure.

Spontaneous MI: Includes myocardial infarction that occurs without procedural intervention, often related to underlying coronary disease progression.

Clinically driven TLR: Defined as revascularization procedures (either balloon angioplasty or stenting) required due to symptoms (e.g., chest pain) or evidence of ischemia (positive stress testing).

Protocol-driven TLR: Revascularization procedures driven by predefined criteria (e.g., restenosis ≥50%) observed during follow-up imaging.

Protocol-driven TVR: Revascularization of the entire target vessel driven by protocol, typically triggered by angiographic evidence of significant restenosis (≥50%).

Independent Adjudication: Indicates whether an independent committee or adjudicator was involved in the endpoint assessment process to ensure unbiased event reporting.

Risk of bias was evaluated using the RoB 2 tool (Figures 2A,B). The randomization process was generally judged as low risk across most trials. However, some concerns were identified regarding deviations from intended interventions and outcome measurement, primarily arising from the open-label design of several included studies.

**Figure 2:**
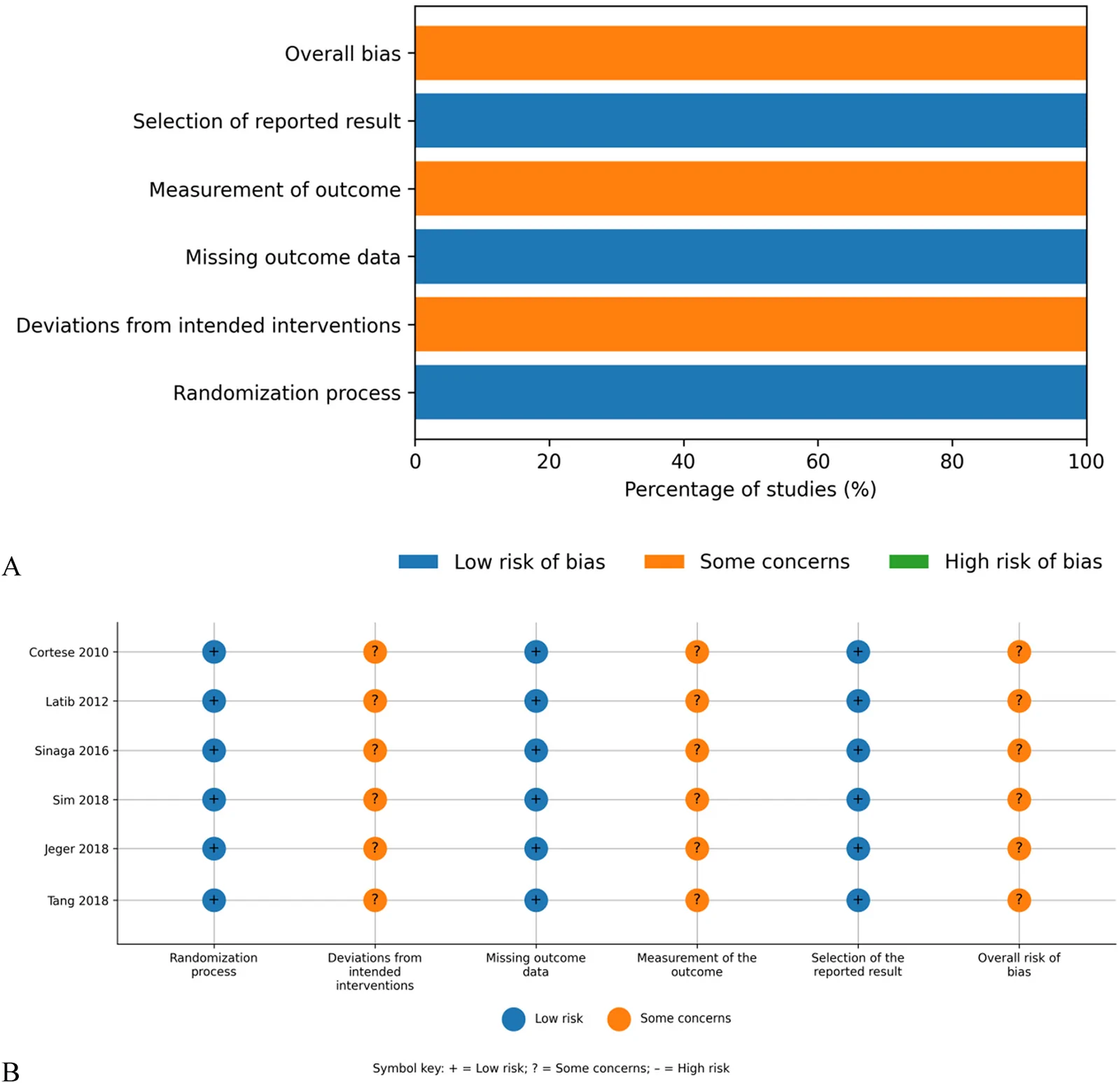
Risk of bias assessment of the included randomized controlled trials using the cochrane RoB 2 tool. **(A)** Summary plot showing the proportion of judgments across each risk-of-bias domain among the included randomized controlled trials; **(B)** traffic light plot showing risk-of-bias judgments for each individual randomized controlled trial.

### 3.3 Nonfatal myocardial infarction

After accounting for overlapping publications from the same RCT cohorts, six unique trials contributed data on nonfatal myocardial infarction. Among 1,849 participants, events occurred in 33 of 875 patients in the DCB group and 42 of 974 in the DES group. Pooled analysis showed no statistically significant difference between groups (OR = 0.87, 95% CI: 0.55–1.39; *I*^2^ = 39%; Figure 3A). Funnel plot inspection did not reveal obvious asymmetry; however, with fewer than 10 trials, this assessment is exploratory and cannot definitively exclude publication bias (Figure 3B).

**Figure 3:**
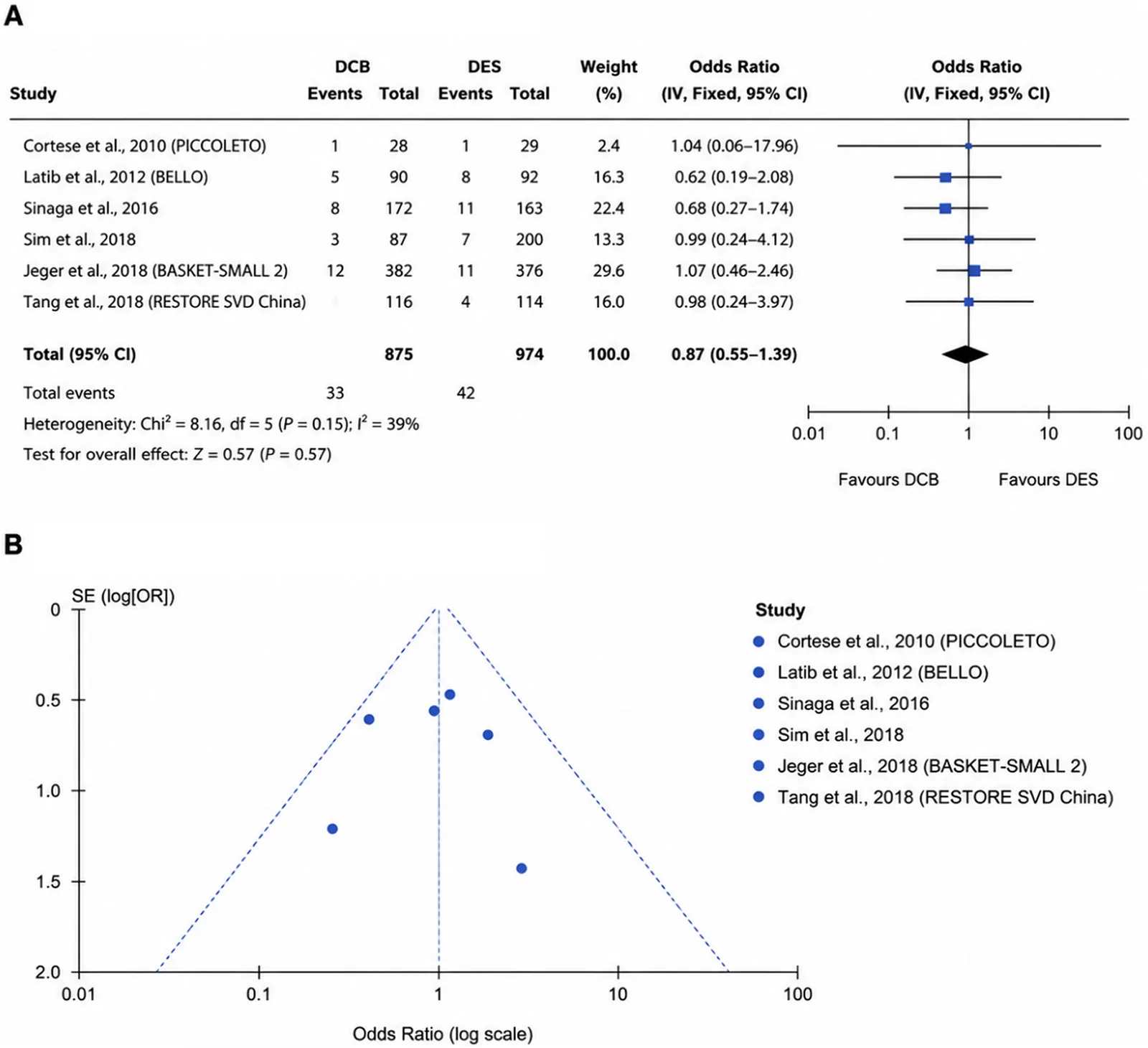
Meta-analysis of nonfatal myocardial infarction comparing drug-coated balloons with drug-eluting stents after accounting for overlapping randomized controlled trial cohorts. **(A)** Forest plot showing pooled odds ratios for nonfatal myocardial infarction. **(B)** Funnel plot showing the distribution of effect estimates. Because fewer than 10 randomized controlled trials contributed to this outcome, funnel plot asymmetry was interpreted as an exploratory assessment rather than definitive evidence of publication bias.

### 3.4 Cardiac death

Cardiac death was reported in six the included RCTs. Using a fixed-effects model, events occurred in 16 of 613 patients in the DCB group and 13 of 719 in the DES group, with no significant difference (OR = 1.38, 95% CI: 0.66–2.88; *I*^2^ = 0%; Figure 4A). The funnel plot (Figure 4B) served only as an exploratory visualization.

**Figure 4:**
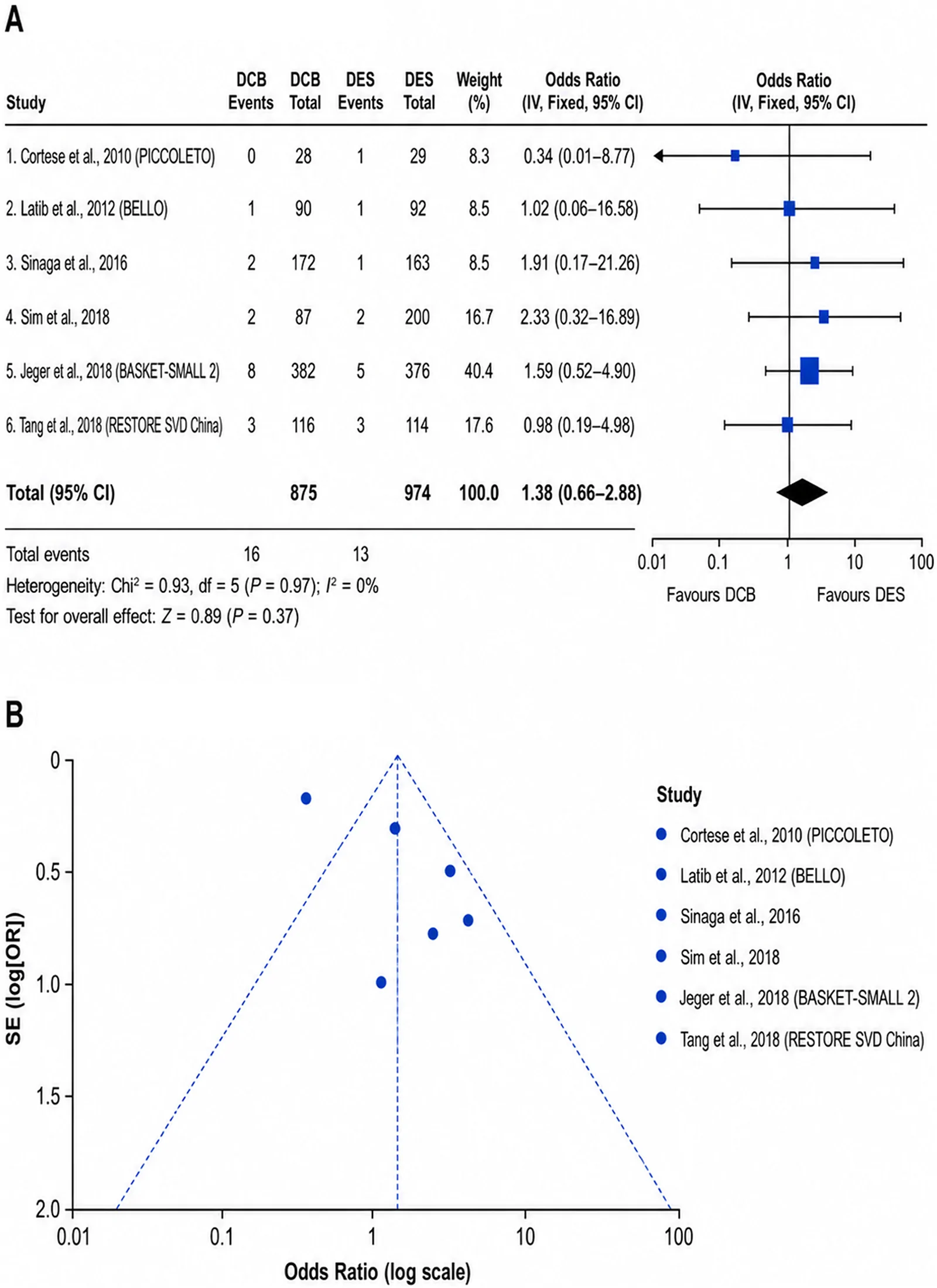
Meta-analysis of cardiac death comparing drug-coated balloons with drug-eluting stents after accounting for overlapping randomized controlled trial cohorts. **(A)** Forest plot showing pooled odds ratios for cardiac death. **(B)** Funnel plot showing the distribution of effect estimates for cardiac death. Because only a limited number of randomized controlled trials contributed to this outcome, funnel plot asymmetry was interpreted cautiously as an exploratory assessment rather than definitive evidence of publication bias.

### 3.5 All-cause death

All-cause death was analyzable from all six RCTs. Heterogeneity was negligible (*I*^2^ = 0%), so a fixed-effects model was applied. No significant difference was observed between DCB and DES (OR = 1.00, 95% CI: 0.59–1.69; Figure 5A). Funnel plot interpretation was exploratory. No substantial funnel plot asymmetry was observed. However, given the limited number of included randomized controlled trials, funnel plot analysis had insufficient statistical power to reliably identify or exclude publication bias.

**Figure 5:**
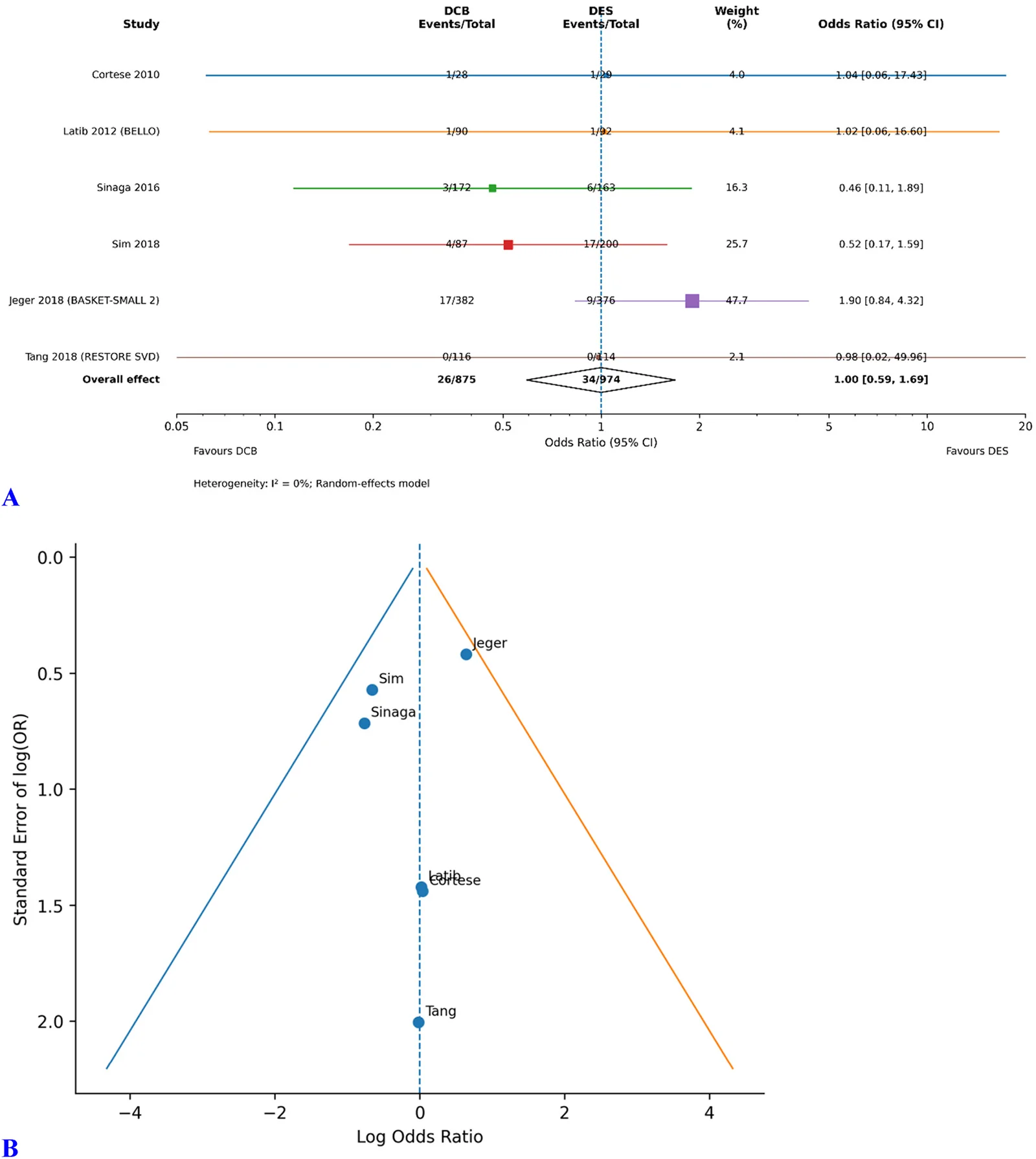
Meta-analysis of all-cause death comparing drug-coated balloons with drug-eluting stents using unique randomized controlled trial cohorts. **(A)** Forest plot showing pooled odds ratios for all-cause death. **(B)** Funnel plot showing the distribution of effect estimates for all-cause death.

### 3.6 Target lesion revascularization

For TLR, six trials provided data. Fixed-effects pooling showed no significant difference between DCB and DES (OR = 1.03, 95% CI: 0.65–1.64; *I*^2^ = 25%; Figure 6A). The funnel plot (Figure 6B) was interpreted exploratorily. No obvious asymmetry was observed in the funnel plot. However, the limited number of included trials prevents a reliable assessment of publication bias.

**Figure 6:**
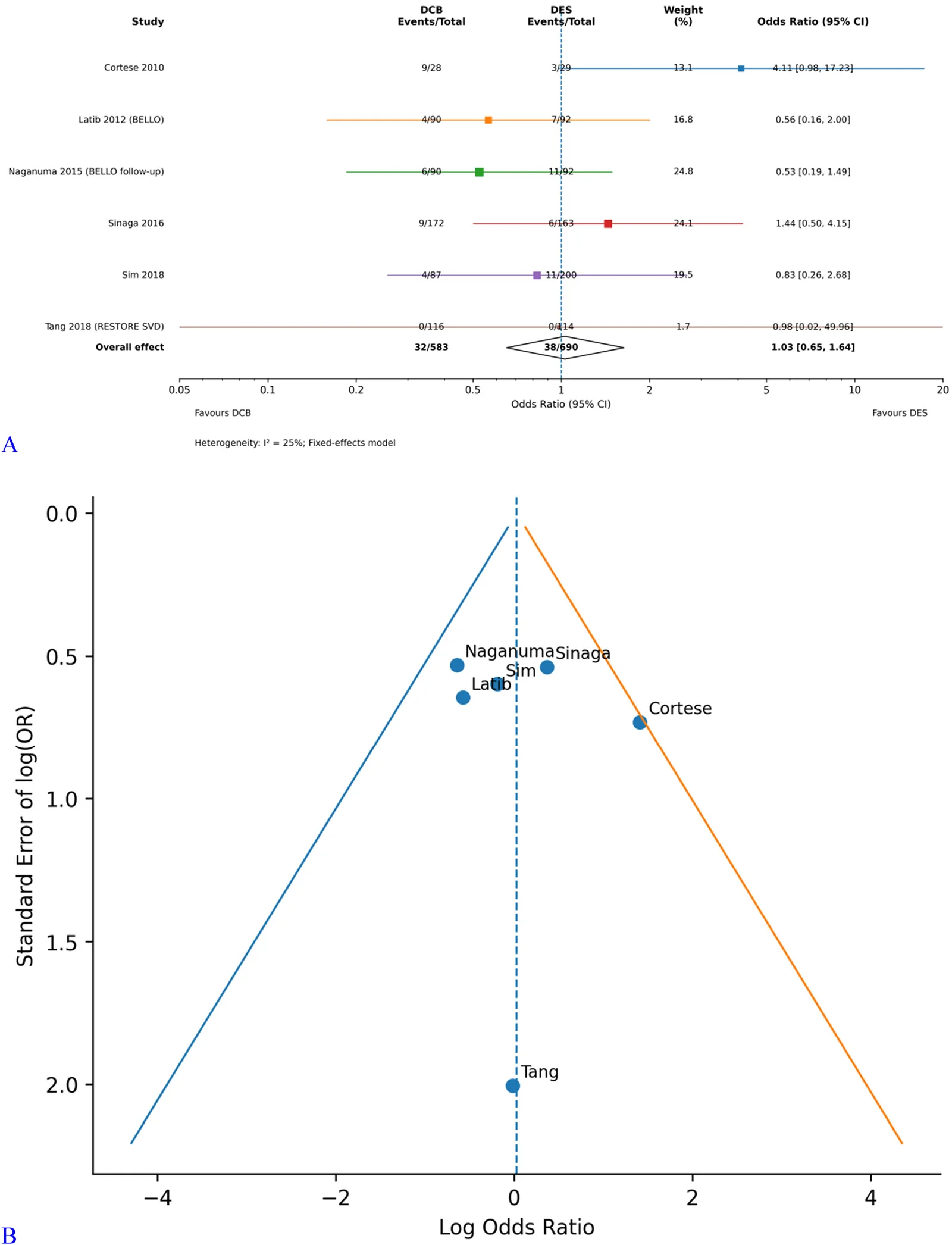
Meta-analysis of target lesion revascularization comparing drug-coated balloons with drug-eluting stents. **(A)** Forest plot showing pooled odds ratios for target lesion revascularization. **(B)** Funnel plot showing the distribution of effect estimates for target lesion revascularization.

### 3.7 Target-vessel revascularization

TVR was evaluated in all six trials. Moderate heterogeneity (*I*^2^ = 34%) prompted use of a random-effects model, which yielded no significant difference (OR = 0.76, 95% CI: 0.47–1.23; Figure 7A). Funnel plot assessment (Figure 7B) was exploratory, as fewer than 10 trials were included.

**Figure 7:**
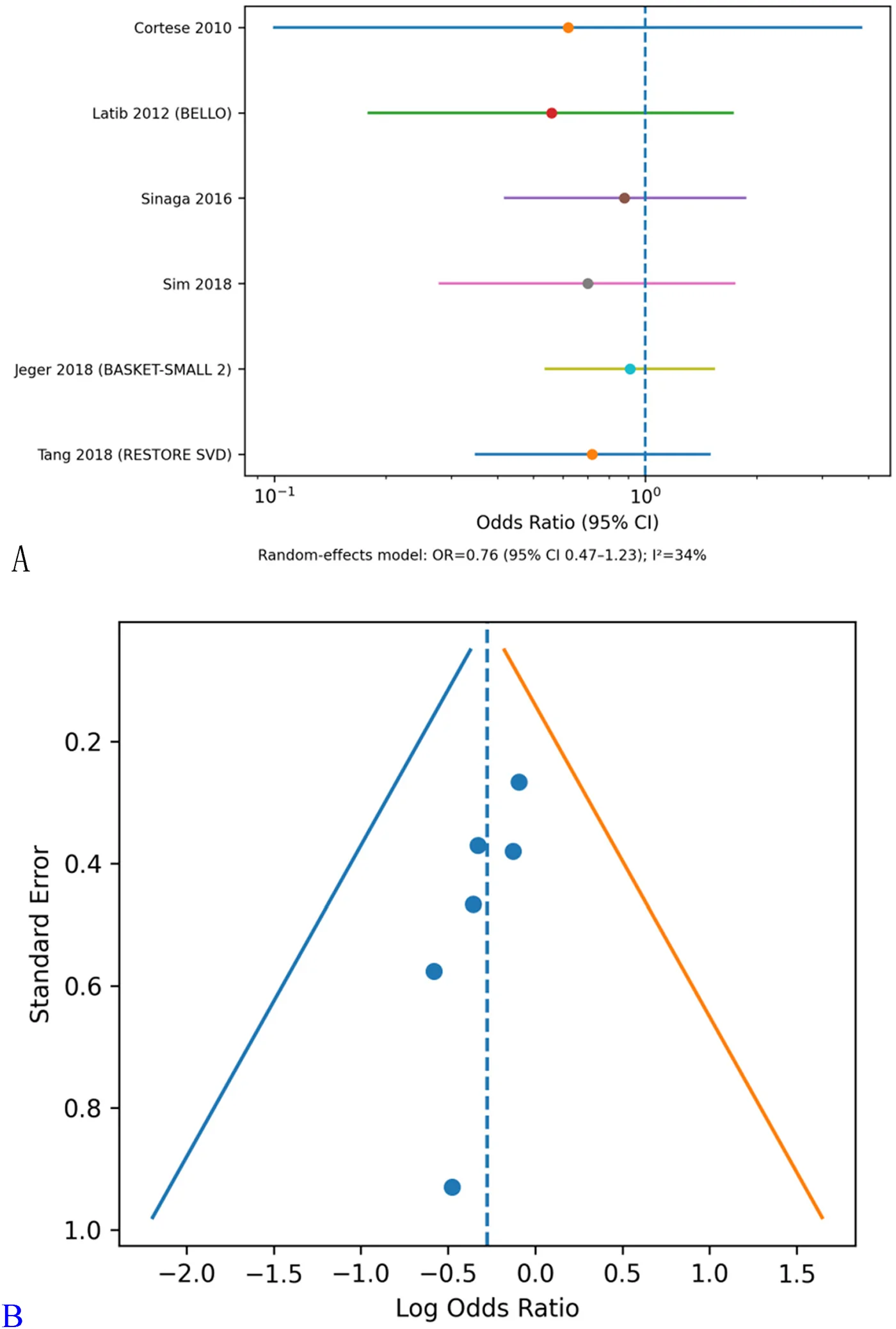
Meta-analysis of target-vessel revascularization comparing drug-coated balloons with drug-eluting stents after accounting for unique randomized controlled trial cohorts. **(A)** Forest plot showing pooled odds ratios for target-vessel revascularization. The random-effects model demonstrated no statistically significant difference between DCB and DES groups (OR = 0.76, 95% CI: 0.47–1.23; *I*^2^ = 34%). **(B)** Funnel plot showing the distribution of effect estimates. No apparent asymmetry was observed; however, because fewer than 10 randomized controlled trials contributed to this outcome, funnel plot interpretation should be considered exploratory rather than definitive evidence of publication bias.

### 3.8 Subgroup and sensitivity analysis

Subgroup analyses by vessel diameter and drug type, as well as sensitivity analyses restricted to 12-month follow-up, were performed to explore heterogeneity and evaluate the robustness of the pooled estimates.

#### 3.8.1 Vessel diameter analysis (RVD Threshold)

Vessels were categorized as “very small” (RVD ≤ 2.25 mm) or “borderline” small (RVD > 2.25 mm). No statistically significant interaction was observed between subgroups (*P* interaction > 0.05), indicating that the overall treatment effect was not significantly modified by vessel size. Although a lower risk of TLR was observed in the very small vessel subgroup (OR = 0.65, 95% CI: 0.45–0.95), no such reduction was seen for TVR (OR = 0.72, 95% CI: 0.50–1.03). These findings should be interpreted cautiously, as subgroup analyses were exploratory and not powered to demonstrate differential treatment effects. Overall, the available evidence does not establish a definitive treatment advantage for any specific vessel-size subgroup.

#### 3.8.2 Device Sensitivity

All included trials evaluated paclitaxel-coated balloons; therefore, subgroup analysis according to drug type was not performed. Excluding the oldest-generation DES trial (PICCOLETO) did not materially alter the pooled estimate for TLR (OR = 1.01, 95% CI: 0.62–1.65).

### 3.9 Certainty of evidence (GRADE assessment)

The certainty of evidence was assessed using the GRADE framework for major clinical outcomes. The certainty of evidence was rated as moderate for nonfatal myocardial infarction, cardiac death, and all-cause death. Evidence certainty for revascularization outcomes was considered moderate for target lesion revascularization and low to moderate for target-vessel revascularization. Downgrading decisions were based on outcome-specific methodological considerations rather than generalized assumptions. For mortality and myocardial infarction outcomes, certainty was mainly reduced because of imprecision related to relatively few accumulated events and wide confidence intervals, which limited exclusion of clinically meaningful benefit or harm. For revascularization outcomes, additional concerns included lack of blinding in several trials, variation in endpoint definitions (clinically driven vs. protocol-driven revascularization), and potential ascertainment bias caused by scheduled angiographic follow-up. Although heterogeneity was generally low, differences in DCB platforms, DES generations, and procedural strategies contributed to indirectness concerns for some comparisons, as summarized in Table 3.

**Table 3:** GRADE assessment of evidence for key outcomes.

Outcome	Certainty of Evidence (GRADE)	Reasons for Downgrading
Nonfatal Myocardial Infarction	Moderate	Downgraded due to imprecision caused by relatively low event numbers and wide confidence intervals, which limited the certainty regarding small differences between DCB and DES.
Cardiac Death	Moderate	Downgraded due to imprecision resulting from the low frequency of cardiac death events and insufficient statistical power to detect clinically meaningful differences, despite consistent effects across studies.
All-Cause Death	Moderate	Downgraded because the number of observed deaths was relatively small, resulting in uncertainty regarding the precision of pooled estimates despite low statistical heterogeneity.
Target Lesion Revascularization	Moderate	Downgraded due to potential risk of outcome assessment bias related to open-label trial designs and variations in endpoint definitions, including differences between clinically driven and protocol-driven revascularization.
Target Vessel Revascularization	Low to Moderate	Downgraded due to multiple sources of uncertainty, including non-blinded outcome assessment, protocol-driven angiographic surveillance in some trials, and heterogeneity in TVR definitions across studies.

## 4 Discussion

Vessel diameter is a critical predictor of restenosis in patients with small coronary artery disease. Percutaneous coronary intervention in this setting presents inherent challenges, including the risk of acute vessel closure due to elastic recoil after conventional balloon angioplasty and the occurrence of neointimal hyperplasia following stent implantation (14). Current guidelines and consensus documents indicate that the optimal therapeutic strategy for *de novo* small coronary artery disease remains undefined. Drug-coated balloons have emerged as a promising therapeutic modality (15). However, similar to traditional balloon angioplasty, DCBs have limitations, such as elastic recoil or dissection, which may necessitate urgent bailout stenting. Clinical outcomes with DCB are highly dependent on lesion preparation standards and bailout stenting thresholds. Variability in these procedural aspects across trials can significantly influence clinical event rates and pooled estimates, underscoring the importance of adhering to international consensus recommendations (16).

Concerns persist regarding whether DCBs can deliver adequate and sustained drug concentrations to the vessel wall compared with DES, given the brief balloon inflation time and potential washout by blood flow (17). While numerous studies have demonstrated the benefits of DCBs in small coronary artery disease, the absence of control groups in some investigations limits the conclusiveness of their findings. Previous meta-analyses have evaluated the comparative effectiveness of DCB and DES in this setting, but differences in included populations, device generations, and methodological approaches may explain the variations among reported results (18). Li and colleagues previously reported comparable clinical outcomes between DCB and DES in *de novo* small vessel disease, although their analysis included fewer randomized trials and earlier-generation devices (1). Later randomized evidence, including the BASKET-SMALL 2 and RESTORE SVD trials, incorporated contemporary DES platforms and standardized DCB protocols, providing more clinically relevant comparisons. Our findings are generally consistent with these prior analyses, showing no significant differences between DCB and DES for myocardial infarction, mortality, TLR, or TVR. The consistency across studies may reflect comparable antiproliferative effects of both technologies in preventing neointimal hyperplasia. Nevertheless, discrepancies in individual outcomes may arise from differences in lesion characteristics, vessel diameter thresholds, DCB drug platforms, bailout stenting criteria, and endpoint definitions. Importantly, our analysis was restricted to randomized controlled trials and accounted for overlapping publications from the same cohorts, which may provide a more conservative estimate of treatment effects.

Our investigation found no significant difference in the risk of nonfatal myocardial infarction between DCB and DES strategies, indicating comparable safety profiles. This finding may be attributable to the absence of a permanent metal scaffold with DCB, which may mitigate late inflammation, promote positive vascular remodeling, and permit shorter durations of dual antiplatelet therapy (19). Furthermore, bailout stent implantation remains a valuable contingency in cases of suboptimal angiographic results, significant dissection, or acute elastic recoil after DCB use. This approach reduces the need for unnecessary routine stent placement and may be more acceptable to patients with small vessel disease (20).

Our analysis also revealed no significant differences between the two treatment strategies with respect to cardiac death, all-cause death, or target vessel revascularization. These findings are consistent with prior research indicating that routine stent implantation does not confer significant improvements in these outcomes for patients with *de novo* small coronary artery disease compared with balloon angioplasty alone. The lack of a definitive advantage for stent implantation in small vessels may relate to vessel diameter itself (21). Small vessels often carry a heightened thrombotic burden, predisposing patients to acute or subacute thrombosis and adverse sequelae after stent placement.

In the treatment of small vessels, DCBs may offer specific advantages over DES by enabling drug delivery without permanent metallic scaffolds and preserving native vascular anatomy (22). However, DCBs represent a heterogeneous intervention class, with differences in drug platforms and coating technologies that may influence outcomes. Paclitaxel remains the most extensively studied antiproliferative agent in coronary DCB technology because of its high lipophilicity, rapid tissue uptake, and prolonged vascular wall retention after brief balloon inflation. Limus-based DCB platforms, including sirolimus-coated balloons, have attracted increasing attention due to their potent inhibition of smooth muscle cell proliferation and potentially improved safety profile, but clinical evidence in *de novo* small coronary lesions remains limited. Whether newer limus-based DCB technologies can provide superior outcomes compared with established paclitaxel-coated balloons remains uncertain and requires dedicated randomized trials.

Our pre-specified subgroup and sensitivity analyses explored whether vessel size, drug type, or follow-up duration influenced treatment effects. Although the very small vessel subgroup (RVD ≤ 2.25 mm) showed a statistically significant reduction in TLR with DCB treatment, no significant interaction was detected between vessel-size categories. This finding should therefore be regarded as hypothesis-generating rather than evidence of a confirmed subgroup-specific benefit. Further adequately powered randomized trials are required to determine whether patients with very small vessels derive greater benefit from DCB therapy compared with DES.

Collectively, our results support DCB as a feasible alternative to DES for patients with *de novo* small coronary artery disease, given their comparable clinical outcomes across key endpoints. Nevertheless, further investigations are warranted to corroborate these findings and to explore the long-term efficacy and safety of DCBs relative to DES in this specific population. The evolution of next-generation DCB technologies and advancements in intravascular imaging hold promise for enhancing the efficacy and applicability of DCB therapy in managing small vessel disease. From a clinical standpoint, DCB represents a reasonable alternative for selected *de novo* small-vessel lesions, provided that optimal lesion preparation is achieved and bailout stenting rates remain low. Clinician decision-making should account for remaining uncertainties, including long-term safety beyond typical trial follow-up periods, the comparative performance of specific DCB drug platforms, and variations in endpoint definitions such as those for myocardial infarction.

Optimizing periprocedural and post-procedural antithrombotic therapy is crucial in small vessel interventions. Intensified strategies, such as intravenous cangrelor for periprocedural platelet inhibition or tailored post-PCI antithrombotic regimens, may further mitigate ischemic risks in this complex patient population (23, 24).

In addition to anatomical characteristics, patient-related factors such as advanced age, diabetes mellitus, and chronic kidney disease may influence restenosis risk and treatment selection in small vessel coronary disease. However, the included randomized trials did not consistently report these clinical characteristics in a manner that allowed reliable subgroup analyses. Therefore, this meta-analysis could not determine whether specific patient populations, including older adults, patients with diabetes, or those with chronic kidney disease, derive greater benefit from DCB compared with DES. Future studies incorporating comprehensive patient-level data are warranted to identify clinically meaningful responder profiles.

Our analysis has several limitations. First, although this meta-analysis was restricted to randomized controlled trials to enhance methodological consistency, the number of included trials remains limited. Moreover, although funnel plots were provided for visual assessment, all outcomes included fewer than 10 trials. Therefore, these plots were insufficiently powered to confirm or exclude publication bias, and their interpretation should remain cautious. The statistical power for detecting differences in relatively infrequent clinical outcomes may be insufficient, and further large-scale multicenter randomized trials are warranted. Second, follow-up duration varied among the included studies, which could affect the assessment of long-term outcomes. Although the included studies exhibited some heterogeneity, we enhanced methodological transparency by using the RoB 2 tool and providing a GRADE assessment of the evidence, offering a clear framework for interpreting the results. Sensitivity analyses restricted to a common 12-month follow-up window confirmed the stability of our pooled estimates, reinforcing the clinical applicability of DCB as a viable alternative to DES despite minor protocol differences. Third, standardized endpoint interpretation remains a challenge, as variability in myocardial infarction definitions and revascularization ascertainment (clinically driven vs. protocol-driven) across studies may introduce uncertainty and affect treatment effect estimates (25). Finally, our analysis focused on clinical efficacy and safety outcomes and did not evaluate cost-effectiveness or quality-of-life measures.

## 5 Conclusion

This meta-analysis demonstrates that drug-coated balloons provide clinical outcomes comparable to drug-eluting stents in patients with *de novo* small coronary artery disease, with no significant differences in myocardial infarction, mortality, or revascularization. DCB represents a reasonable alternative to DES when optimal lesion preparation is achieved.

Several uncertainties remain. The limited number of trials constrains statistical power, and the exploratory subgroup findings require confirmation in dedicated studies. Evidence is insufficient to guide treatment selection for high-risk subgroups, including older adults, diabetics, and patients with chronic kidney disease.

Future adequately powered randomized trials with standardized endpoints and longer follow-up are needed to refine patient selection. Until then, treatment decisions should be individualized based on lesion characteristics, comorbidities, and operator expertise.

## Data Availability

The original contributions presented in the study are included in the article/Supplementary Material, further inquiries can be directed to the corresponding author/s.

## References

[1] Li M Guo C Lv YH Zhang MB Wang ZL. Drug-coated balloon versus drug-eluting stent in *de novo* small coronary vessel disease: a systematic review and meta-analysis. Medicine (Baltimore). (2019) 98(21):e15622. 10.1097/MD.0000000000015622 31124941 PMC6571399

[2] Puymirat E Mangiacapra F Peace A Ntarladimas Y Conte M Bartunek J. Safety and effectiveness of drug-eluting stents versus bare-metal stents in elderly patients with small coronary vessel disease. Arch Cardiovasc Dis. (2013) 106(11):554–61. 10.1016/j.acvd.2013.06.056 24231052

[3] Yerasi C Case BC Forrestal BJ Torguson R Weintraub WS Garcia-Garcia HM. Drug-coated balloon for *de novo* coronary artery disease: JACC state-of-the-art review. J Am Coll Cardiol. (2020) 75(9):1061–73. 10.1016/j.jacc.2019.12.046 32138967

[4] Merinopoulos I Gunawardena T Corballis N Bhalraam U Reinhold J Wickramarachchi U. Assessment of paclitaxel drug-coated balloon only angioplasty in STEMI. JACC Cardiovasc Interv. (2023) 16(7):771–9. 10.1016/j.jcin.2023.01.380 37045498

[5] Muramatsu T Kozuma K Tanabe K Morino Y Ako J Nakamura S. Clinical expert consensus document on drug-coated balloon for coronary artery disease from the Japanese association of cardiovascular intervention and therapeutics. Cardiovasc Interv Ther. (2023) 38(2):166–76. 10.1007/s12928-023-00921-2 36847902 PMC10020262

[6] De La Torre Hernandez J Puri R Alfonso F. Drug-coated balloon: “scoring to win”. JACC Cardiovasc Interv. (2017) 10(13):1341–3. 10.1016/j.jcin.2017.05.019 28683940

[7] Banerjee S Khalili H. Drug-coated balloon for long femoropopliteal lesions. Circ Cardiovasc Interv. (2018) 11(10):e7084. 10.1161/CIRCINTERVENTIONS.118.007084 30354645

[8] Cortese B Micheli A Picchi A Coppolaro A Bandinelli L Severi S. Paclitaxel-coated balloon versus drug-eluting stent during PCI of small coronary vessels, a prospective randomised clinical trial. The PICCOLETO study. Heart. (2010) 96:1291–6. 10.1136/hrt.2010.195057 20659948

[9] Latib A Colombo A Castriota F Micari A Cremonesi A De Felice F. A randomized multicenter study comparing a paclitaxel drug-eluting balloon with a paclitaxel-eluting stent in small coronary vessels: the BELLO (Balloon elution and late loss optimization) study. J Am Coll Cardiol. (2012) 60:2473–80. 10.1016/j.jacc.2012.09.020 23158530

[10] Sinaga DA Ho HH Watson TJ Sim A Nyein TT Jafary FH. Drug-coated balloons: a safe and effective alternative to drug-eluting stents in small vessel coronary artery disease. J Interv Cardiol. (2016) 29:454–60. 10.1111/joic.12333 27578540

[11] Sim HW Ananthakrishna R Chan SP Low AF Lee CH Chan MY. Treatment of very small *de novo* coronary artery disease with 2.0 mm drug-coated balloons showed 1-year clinical outcome comparable with 2.0 mm drug-eluting stents. J Invasive Cardiol. (2018) 30(7):256–61. 29656281

[12] Jeger RV Farah A Ohlow M-A Mangner N Möbius-Winkler S Leibundgut G. Drug-coated balloons for small coronary artery disease (BASKET-SMALL 2): an open-label randomised non-inferiority trial. Lancet (London, England). (2018) 392:849–56. 10.1016/S0140-6736(18)31719-7 30170854

[13] Tang Y Qiao S Su X Chen Y Jin Z Chen H. Drug-coated balloon versus drug-eluting stent for small-vessel disease: the RESTORE SVD China randomized trial. JACC Cardiovasc Interv. (2018) 11(23):2381–92. 10.1016/j.jcin.2018.09.009 30522667

[14] Colleran R Fitzgerald S Byrne RA. Coronary drug-coated balloon angioplasty: what are the next steps? JACC Cardiovasc Interv. (2023) 16(7):768–70. 10.1016/j.jcin.2023.01.382 37045497

[15] Hawkins BM Meier IC. Drug-coated balloon, limb ischemia, and the elephant in the room. JACC Cardiovasc Interv. (2020) 13(19):2300–2. 10.1016/j.jcin.2020.07.036 32950414

[16] Jeger RV Eccleshall S Wan Ahmad WA Ge J Poerner TC Shin E-S. Drug-coated balloons for coronary artery disease: third report of the international DCB consensus group. JACC Cardiovasc Interv. (2020) 13(12):1391–402. 10.1016/j.jcin.2020.02.043 32473887

[17] Sattar Y Aronow HD Alam M. Drug-coated balloon failure following femoro-popliteal intervention: where to draw the line? J Am Coll Cardiol. (2022) 80(13):1251–3. 10.1016/j.jacc.2022.08.002 36137675

[18] Ghobrial J. Drug-coated balloon angioplasty in pulmonary vein stenosis: a promising tool. JACC Case Rep. (2023) 24:102021. 10.1016/j.jaccas.2023.102021 37869220 PMC10589446

[19] Liistro F. Is drug-coated balloon the real winner for femoropopliteal artery intervention? JACC Cardiovasc Interv. (2023) 16(13):1651–3. 10.1016/j.jcin.2023.05.048 37438032

[20] Nerla R Castriota F Cremonesi A Micari A. Stellarex drug-coated balloon: a technology evaluation. Expert Opin Drug Deliv. (2020) 17(9):1181–8. 10.1080/17425247.2020.1787982 32615811

[21] Zhao H Miao R Lin F Zhao G. Drug-coated balloon in primary percutaneous coronary intervention. J Interv Cardiol. (2023) 2023:5210808. 10.1155/2023/5210808 37404481 PMC10317576

[22] Sachdeva B Abreo K. Drug-coated balloon angioplasty for hemodialysis fistula maintenance. Clin J Am Soc Nephrol. (2018) 13(8):1140–1. 10.2215/CJN.07360618 30042226 PMC6086701

[23] Gargiulo G Cirillo P Sperandeo L Castiello DS Manzi L Forzano I. Pharmacodynamic effects of cangrelor in patients with acute or chronic coronary syndrome undergoing percutaneous coronary intervention: the POMPEII registry. EuroIntervention. (2025 May 16) 21(10):560–70. 10.4244/EIJ-D-24-00757 PMC12063553 40375762

[24] Castiello DS Oliva A Andò G Niccoli G Pelliccia F Moscarella E. Antithrombotic therapy in complex percutaneous coronary intervention. EuroIntervention. (2025) 21(18):e1051–68. 10.4244/EIJ-D-24-00992 40958619 PMC12418109

[25] Fezzi S Scheller B Cortese B Alfonso F Jeger R Colombo A. Definitions and standardized endpoints for the use of drug-coated balloon in coronary artery disease: consensus document of the drug coated balloon academic research consortium. Eur Heart J. (2025) 46(26):2498–519. 10.1093/eurheartj/ehaf029 40270117 PMC12232911

